# Reliability Simulation Analysis of TSV Structure in Silicon Interposer under Temperature Cycling

**DOI:** 10.3390/mi15080986

**Published:** 2024-07-30

**Authors:** Wenchao Tian, Haojie Dang, Dexin Li, Yunhao Cong, Yuanming Chen

**Affiliations:** 1 School of Electro-Mechanical Engineering, Xidian University, Xi’an 710000, China; d2100710345@outlook.com (H.D.); 22041212901@stu.xidian.edu.cn (D.L.); congyunhao2021@163.com (Y.C.); 2 State Key Laboratory of Electromechanical Integrated Manufacturing of High-Performance Electronic Equipments, Xidian University, Xi’an 710000, China; 3 Shanghai Sharetek Technology Co., Ltd., Shanghai 201109, China; frankchen@sharetek.com.cn

**Keywords:** multi-layered interposers, electroplating defect, temperature cycling, reliability

## Abstract

As semiconductor integration scales expand and chip sizes shrink, Through Silicon Via (TSV) technology advances towards smaller diameters and higher aspect ratios, posing significant challenges in thermo-mechanical reliability, particularly within interposer substrates where mismatched coefficients of thermal expansion exacerbate issues. This study conducts a thermo-mechanical analysis of TSV structures within multi-layered complex interposers, and analyzes the thermal stress behavior and reliability under variable temperature conditions (−55 °C to 85 °C), taking into account the typical electroplating defects within the copper pillars in TSVs. Initially, an overall model is established to determine the critical TSV locations. Sub-model analysis is then employed to investigate the stress and deformation of the most critical TSV, enabling the calculation of the temperature cycle life accordingly. Results indicate that the most critical TSV resides centrally within the model, exhibiting the highest equivalent stress. During the temperature cycling process, the maximum deformation experiences approximately periodic variations, while the maximum equivalent stress undergoes continuous accumulation and gradually diminishes. Its peak occurs at the contact interface corner between the TSV and Redistribution Layer (RDL). The estimated life of the critical point is 3.1708 × $10^{5}$ cycles. Furthermore, it is observed that electroplating defect b alleviates thermal stress within TSVs during temperature cycling. This study provides insights into TSV thermal behavior and reliability, which are crucial for optimizing the design and manufacturing processes of advanced semiconductor packaging.

## 1. Introduction

The inception of TSV technology marked a significant advancement in semiconductor interconnection methodologies. TSV technology revolutionized chip-to-chip intercommunication by establishing vertical pathways between wafers, thereby enabling 2.5D/3D packages and chiplet architectures. With the deceleration of Moore’s Law, there has been a pronounced shift towards advanced packaging technologies to circumvent the limitations posed by traditional scaling trends [1]. TSV emerges as a pivotal solution, facilitating enhanced integration, reduced signal latency, lowered capacitance and inductance, and heightened power efficiency and data transfer rates through its vertical interconnects [2,3,4,5].

The myriad advantages of TSV technology encompass heightened integration density, superior electrical performance, multifunctional integration, and cost-effective manufacturing with high yields. Presently, the drive towards increased integration and minimized chip dimensions propels TSV development towards smaller diameters and heightened depth-to-width ratios. However, the reliability of advanced packaging faces substantial challenges, particularly concerning the thermo-mechanical intricacies inherent in TSV structures [6,7,8,9]. There are many studies aiming to reduce temperature and thermal stress through forced convection methods. Among these, microchannel heat sinks are particularly well suited due to their high surface-area-to-volume ratio, micro-sized dimensions, and compatibility with 3D packaging. Enhancements such as the incorporation of ribs, dimpled surfaces, pin fins, and dimples within the microchannels are used to achieve better heat transfer coefficients [10,11,12,13,14]. While these methods can lower temperatures and alleviate thermal stress gradients, which cannot be completely eliminated, theoretical research remains indispensable. Mechanical stresses in TSV structures primarily stem from two sources. Firstly, the differential coefficients of thermal expansion between the copper-filled TSV and the surrounding silicon substrate induce thermal stresses during temperature fluctuations. These stresses manifest in issues such as TSV expansion, delamination at the copper/insulation interface, and cracks at the TSV-RDL interface [15,16]. Secondly, mechanical stresses arise from TSV plating and annealing processes, exacerbating the formation of voids and cracks within the TSV structure [17,18]. Previous studies have primarily focused on thermal stress analysis of Cu in silicon substrates, with limited applicability due to a lack of research on the influence of additional buffer layers. These studies have only considered stress analysis under fixed-temperature or single-cycle conditions [19,20,21,22]. Therefore, this study considered a complex structure with supplementary layers, including RDL, PI (Polyimide), UBM (Under-Bump Metallization), and SiO_2_ layers to better reflect actual conditions. Additionally, to investigate the impact of temperature variations in practical applications, this study employed cyclic high- and low-temperature conditions. The study also accounted for void defects introduced during the TSV fabrication process.

To address the above issues, this study employed finite element analysis to conduct thermal stress analysis of TSVs within multi-layered complex interposer structures under cyclic temperature variations. Additionally, thermal stress analysis was considered for TSVs containing copper with typical defects. Section 2 describes the numerical method in detail, encompassing geometric configurations, material properties, and boundary conditions. Building upon this foundation, Section 3 presents the result and discussion, systematically conducting stress analysis from three perspectives: the overall model, sub-models, and copper with electroplating defects in TSVs.

## 2. Numerical Methods

### 2.1. Model Geometry and Numerical Scheme

**Figure 1:**
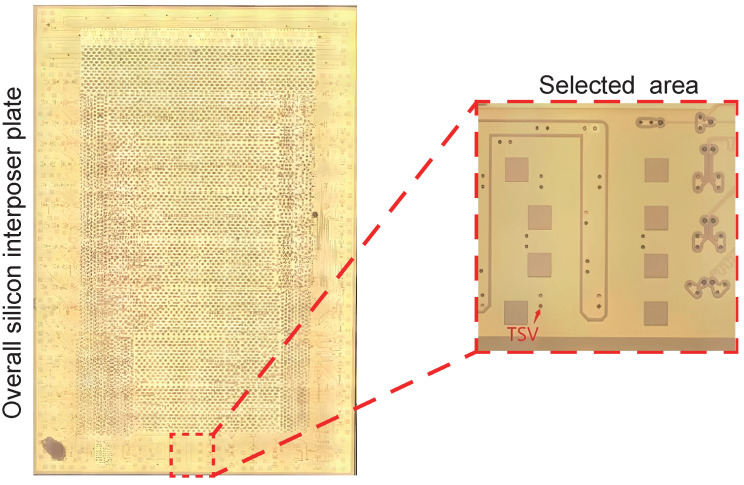
Diagram of overall interposer plate including the selected area.

The dimension of the overall interposer plate is 23 mm × 15 mm. To optimize computational resources without compromising simulation accuracy, only a typical region of 2700 μm × 2700 μm in the interposer plate is selected for 3D modeling. This selected study area is visually indicated in Figure 1 by the red box.

**Figure 2:**
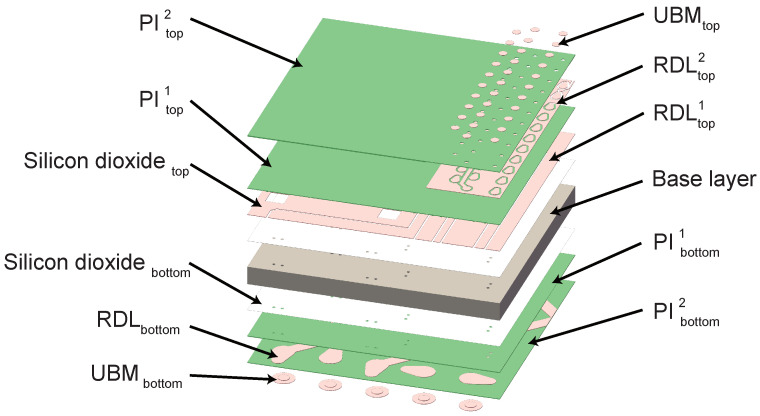
Exploded-view diagram of 3D model.

The structure of the interposer plate is intricately designed. Besides the TSV within the silicon base layer, there are 11 supplementary layers enveloping the base layer. These layers include the RDL layer, PI layer, SiO_2_ layer, and UBM layer, contributing to the comprehensive functionality of the interposer. An exploded view of the established 3D model is portrayed in Figure 2, providing insight into the intricate internal structure of the interposer. The supplementary layers situated above and below the base layer are labeled as “top” and “bottom”, respectively, with each supplementary layer named using subscript notation; detailed descriptions can be found in Table 1.

**Figure 3:**
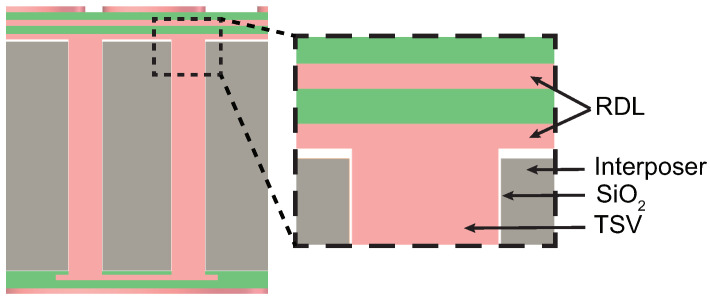
The cross-sectional view of modeling with the TSV dual-hole configuration.

A cross-sectional view of the 3D model with the TSV structure is detailed in Figure 3. Within the silicon base layer, the TSV adopts a dual-hole configuration, with a diameter of 30 μm and a depth-to-width ratio of 7:1. The TSV structure consists of multiple layers, including a dielectric layer, a blocking layer, a seed layer, and a metallic copper filler. However, for the sake of modeling simplicity, the thin blocking and seed layers are streamlined, leaving only the SiO_2_ dielectric layer with a thickness of 0.5 μm.

**Figure 4:**
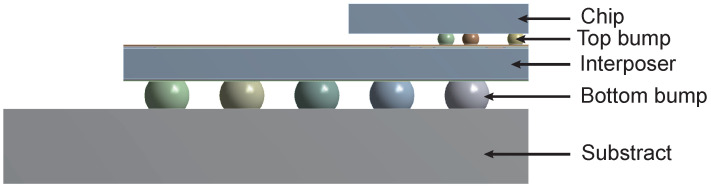
The overall model for finite element simulation.

To simulate real application conditions of the interposer plate and facilitate subsequent boundary condition setups, additional structures including the chip, substrate, top microbumps, and bottom microbumps are incorporated into the model. The overall model for finite element simulation is shown in Figure 4.

### 2.2. Material Parameters

The thermal stress simulation under temperature cycling conditions involves six crucial material parameters, density ρ, Young’s modulus *E*, Poisson’s ratio ν, coefficient of thermal expansion α, coefficient of thermal conductivity κ, and specific heat capacity *C*, as outlined in Table 2. Furthermore, to investigate the fatigue failure of TSV structures under temperature cycling conditions, the Multilinear Isotropic Hardening model is employed to characterize the elasticity and plasticity of the Cu-TSV structures. This model is specifically tailored to address small strain issues under cyclic loading [23], with its corresponding material parameters detailed in Table 3.

### 2.3. Meshing of Models

**Figure 5:**
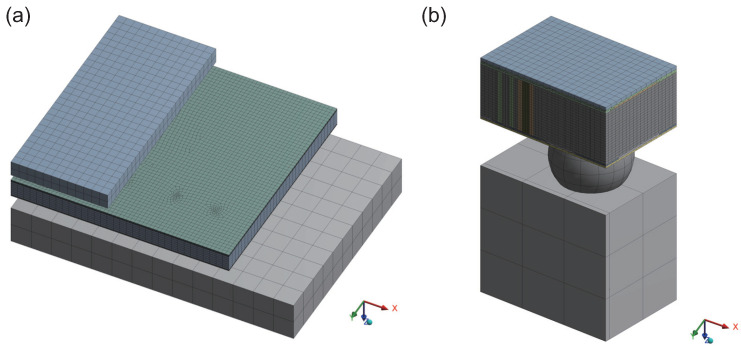
Meshing details of the model: (**a**) the overall model, and (**b**) the sub-model.

To balance computational efficiency and accuracy, this study employed a multi-region meshing strategy. The detailed meshing results of the overall model and sub-models are shown in Figure 5. The average mesh quality is 0.86091. In the sub-model, the TSV structures in the interposer were dense and a key focus of this study, so the mesh was refined. To facilitate observation, the sub-model mesh was sectioned to reveal internal details.

### 2.4. Boundary Conditions

**Figure 6:**
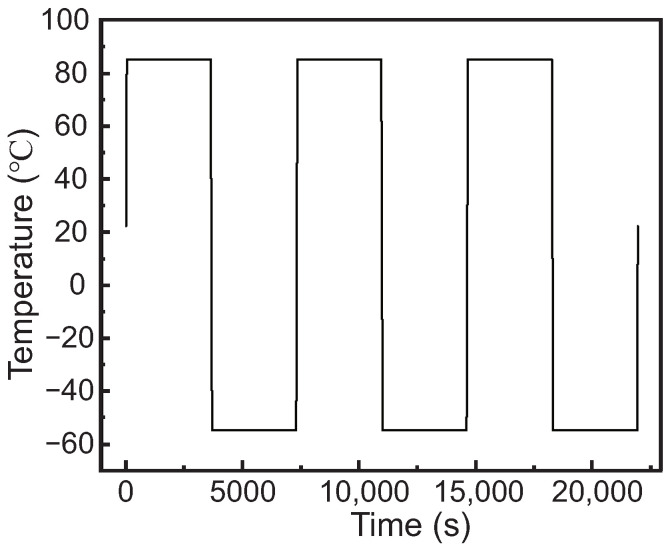
The loading thermal conditions with temperature cycling.

This study employs temperature cycling conditions, starting at environmental temperature (22 °C), as shown in Figure 6. Temperature cycling conditions are employed to simulate thermal stress variations in real-world environments, accelerating failure processes and uncovering product reliability mechanisms in a shorter timeframe. This accelerated failure process, combined with a redundant design, further boosts the system’s fault tolerance and reliability. The high and low temperatures of the temperature cycling are selected according to the JESD22-A104 temperature cycling standard, specifically Test Condition A. The temperature then rises to 85 °C and remains constant for 1 h, followed by a cooling phase to −55 °C, also maintained for 1 h. The transition between high and low temperatures occurs within a minute, and this cycle repeats three times. In the transient temperature module, the temperature load is divided into 13 load steps, each comprising 20 sub-steps.

**Figure 7:**
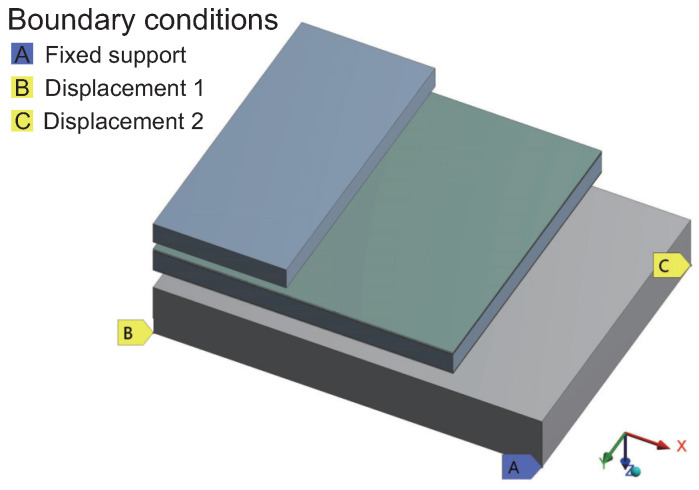
Constraint boundary conditions.

Using 3-point constraints to fix the model allows for minimal constraint while providing freedom of deformation, as illustrated in Figure 7. Point A adopts the “Fix Support” constraint, restricting freedom in the X, Y, and Z directions. Points B and C adopt the “Displacement” constraint, with B limiting freedom in the Y and Z directions, and C limiting freedom only in the Z direction.

## 3. Results and Discussion

### 3.1. Stress Analysis of Overall Model

**Figure 8:**
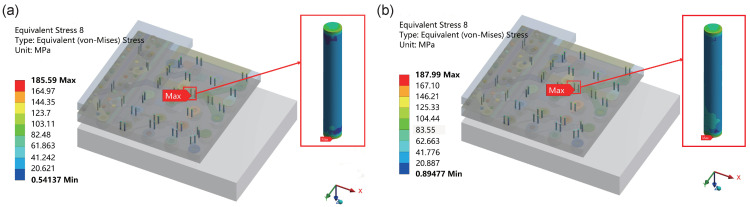
Equivalent stress distribution of TSV in the final cycle on the overall model, where (**a**) represents the high-temperature phase at 85 °C, and (**b**) represents the low-temperature phase at −55 °C.

In the temperature cycling simulation, three cycles of high and low temperatures were conducted, and the deformation of the TSV in the final cycle was analyzed. During temperature cycling, materials and structures typically undergo fatigue and stress accumulation effects after multiple cycles. The stress distribution in the last cycle is crucial for assessing the stability and durability of the TSV structure. Therefore, stress analysis was conducted on the final cycle in this study to more accurately evaluate the performance of the TSV under long-term use or extreme temperature conditions. Figure 8 illustrates the stress distribution of the TSV on the overall model. Figure 8a depicts the equivalent stress distribution during the high-temperature phase of the final cycle, whereas Figure 8b illustrates the equivalent stress distribution during the low-temperature phase of the final cycle.

The results reveal that the maximum equivalent stress on the TSV structure is 185.59 MPa during the high-temperature phase and 187.99 MPa during the low-temperature phase. The equivalent stress during the low-temperature phase is slightly higher than that during the high-temperature phase, and the TSV at the center position of the model consistently experiences the highest equivalent stress, indicating it as the most critical TSV in the model.

Therefore, the TSV with the maximum equivalent stress is located at the center position, which is the most prone to failure. Hence, this most critical TSV in the model is also the focal area of this study. Subsequently, the sub-model approach was selected to conduct detailed research on the most critical TSV, exploring the stress and deformation distribution within the most critical TSV to evaluate the weak failure locations in the interposer.

### 3.2. Deformation and Stress Analysis of Sub-Model

**Figure 9:**
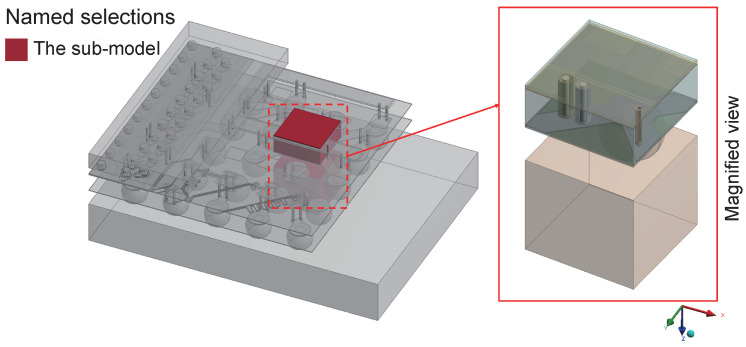
The study area of the sub-model.

The most critical TSV and its surrounding structures were partitioned into an independent sub-model region, and the simulation results from the overall model were used to establish the sub-model. Figure 9 clearly illustrates the schematic diagram of the partitioned sub-model region, with the red area indicating the sub-model.

**Figure 10:**
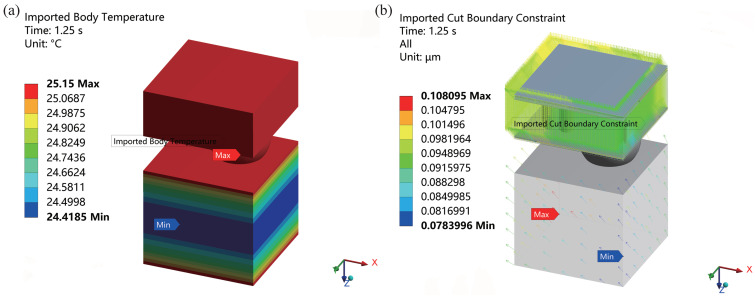
The conditions of the sub-model from the overall model: (**a**) the temperature loading conditions and (**b**) the boundary conditions.

The temperature field of the overall model is utilized as the thermal loading conditions for the sub-model, while the displacement outcomes from the overall model are imposed as boundary conditions of the sub-model. This ensures consistency in temperature conditions between the sub-model and the overall model, facilitating an accurate simulation of the impact of temperature cycling on the TSV structure. Figure 10a illustrates the temperature loading conditions of the sub-model, derived from the temperature field on the segmentation surface of the overall model. Figure 10b depicts the boundary conditions of the sub-model, which are determined by the displacement results from the segmentation surface of the overall model.

During temperature cycling loading, TSV structures expand on both sides due to the thermal expansion coefficient mismatch. Significant stresses occur at the Cu/Si interface, Si/SiO_2_ interface, and TSV/RDL interface, often leading to premature failure and limiting reliability.

**Figure 11:**
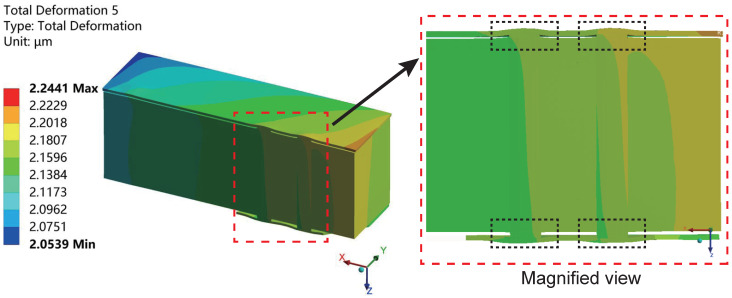
The deformation distribution cross-section along the X-axis of the interposer in the sub-model during the high-temperature stage in the last cycle, with the magnified view highlighted within the red dashed box. The copper expands in the directions along the via, squeezing other supplementary layers.

Figure 11 illustrates the deformation distribution of the interposer in the sub-model during the high-temperature stage in the last cycle. It is evident that due to the significantly higher thermal expansion coefficient of copper compared to silicon, a severe thermal mismatch phenomenon occurs. At high temperatures, the copper expands and extrudes in both directions along the via, which is similar to the result obtained when only considering the copper pillars and silicon base layer with a 2 μm thick SiO_2_ layer added to the top surfaces of both materials [19]. With copper’s thermal expansion coefficient being approximately five times greater than that of silicon, copper is more prone to expansion than the surrounding silicon as temperatures rise, leading to its extrusion from the TSV, referred to by the black dashed box outlined. Additionally, impacted by the PI, RDL, and UBM layers, the extrusion deformation of copper in the TSV exhibits asymmetry. The deformation of the copper pillars within the TSV also results in significant bending deformation of the RDL layer away from the TSV.

**Figure 12:**
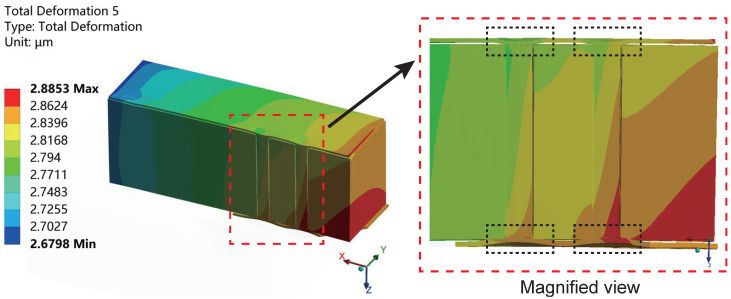
The deformation distribution cross-section along the X-axis of the interposer in the sub-model during the low-temperature stage in the last cycle, with the magnified view highlighted within the red dashed box. The copper shrinks in the directions along the via.

Figure 12 displays the deformation distribution of the interposer in the sub-model during the low-temperature holding stage in the last cycle. Compared to the high-temperature stage, copper contracts more rapidly at low temperatures, causing it to shrink inward along the TSV via direction, with noticeable deformation still present in the RDL layer.

**Figure 13:**
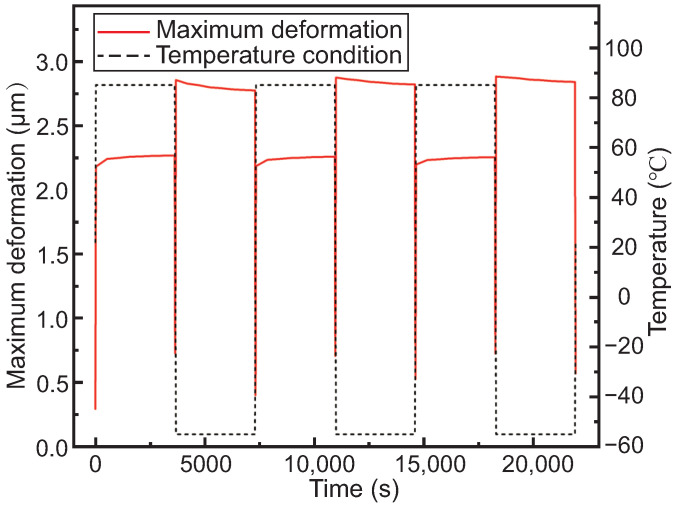
The maximum deformation curve of the interposer in the sub-model with temperature cycling.

Figure 13 presents the curve of the maximum deformation of the interposer in the sub-model over time. It can be observed that within three cycles, the maximum deformation exhibits approximately periodic variation with temperature and has little increase with the number of cycles. Meanwhile, the contraction deformation during the low-temperature stage is greater than the expansion deformation during the high-temperature stage. This periodic deformation indicates a close correlation between deformation and temperature, independent of the cycle number. Once the model structure is determined, the deformation *L*, expressed as L=f(ΔT,Δα), is independent of the time *t*. The greater contraction deformation during the low-temperature stage compared to the expansion deformation during the high-temperature stage may be attributed to the difference in temperature. At an initial temperature of 22 °C, the temperature difference is 63 °C during the high-temperature stage (at 85 °C), while it is 77 °C during the low-temperature stage (at −55 °C).

**Figure 14:**
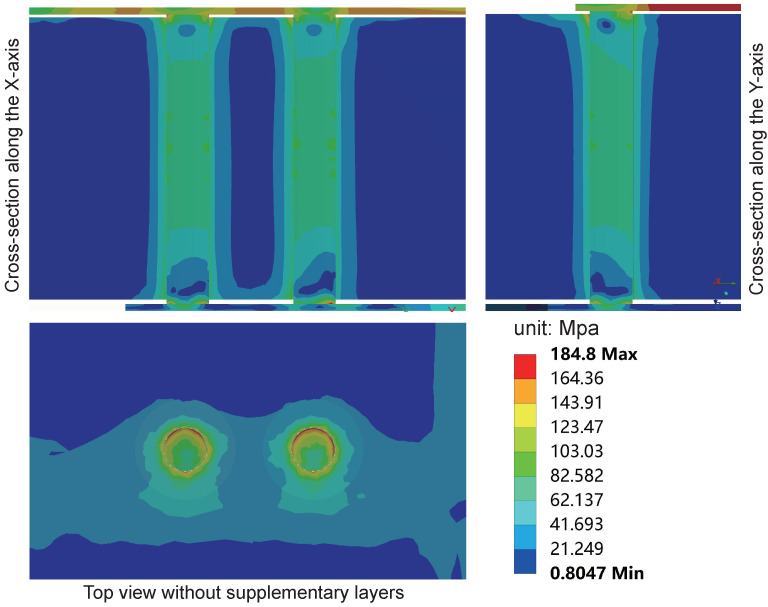
The stress distribution of the interposer in the sub-model during the high-temperature stage at 85 °C in the last cycle. The maximum equivalent stress occurs at the corners of the TSV and RDL contact interface.

**Figure 15:**
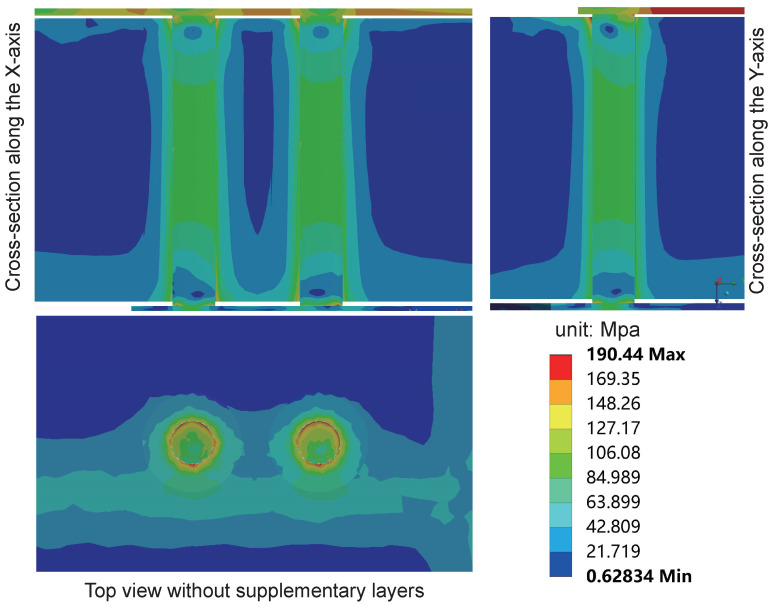
The stress distribution of the interposer inthe sub-model during the low-temperature stage at −55 °C in the last cycle. The maximum equivalent stress occurs at the corners of the TSV and RDL contact interface.

Figure 14 illustrates the equivalent stress distribution on the interposer during the final cycle of the high-temperature stage in the sub-model, while Figure 15 depicts the distribution of equivalent stress during the low-temperature stage. In both high and low-temperature stages, the maximum equivalent stress occurs at the corners of the TSV and RDL contact interface. The maximum equivalent stress during the high-temperature stage is 184.8 MPa, slightly lower than the 190.44 MPa recorded during the low-temperature stage. Additionally, stress concentration is observed at the corners of the Cu/SiO_2_/Si interface near the TSV ends, consistent with previous research findings. This phenomenon may lead to cohesive cracking of the SiO_2_ layer and interfacial cracking of the Cu/SiO_2_ interface, raising reliability concerns. The equivalent stress within the silicon substrate around the vias also significantly increases. The equivalent stress around the Cu/SiO_2_/Si interface is approximately 100 MPa, decreasing gradually to below 20 MPa with increasing distance from the contact interface on the silicon substrate. To better observe the stress distribution in the top view of the copper pillars, supplementary layers above the copper pillars were removed, as detailed in the top views. The results reveal that the equivalent stress on the copper pillars differs from the radial isotropy reported in previous studies [20,21,22], primarily due to the absence of consideration for the PI, UBM, and RDL layers.

**Figure 16:**
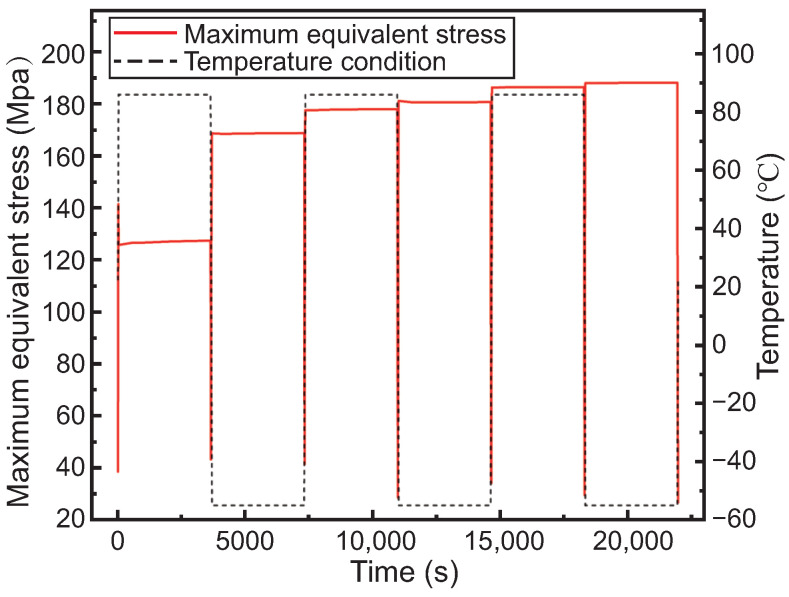
The maximum equivalent stress curve of the interposer in the sub-model with temperature cycling.

Figure 16 depicts the curve of the maximum equivalent stress of the interposer under temperature cycling conditions in the sub-model. In the first cycle, as the temperature rises, the maximum equivalent stress rapidly increases to approximately 140 MPa. This could be attributed to the initial heating stage, where an increase in molecular spacing within the material leads to its volumetric expansion, causing localized stress concentration and consequently a rapid rise in maximum equivalent stress. Subsequently, during the high-temperature hold stage, the maximum stress quickly decreases to around 125 MPa. This reduction may result from the material reaching a stable thermal equilibrium state, where internal thermal stresses are released or material deformation occurs under high-temperature conditions, leading to stress reduction.

Entering the low-temperature stage, the maximum equivalent stress first decreases and then rapidly increases to around 165 MPa. This phenomenon may be attributed to the cooling contraction of the material as the temperature drops, causing structural shrinkage and stress reduction. However, further temperature decrease causes significant inward shrinkage of the copper pillars along the TSV via direction, resulting in increased stress.

In subsequent cycles, the maximum equivalent stress gradually increases, albeit with a diminishing rate of increase. By the third cycle, the maximum equivalent stress reaches approximately 190 MPa. This may be attributed to the cumulative effects of cooling hardening and plastic deformation induced by cyclic loading, leading to internal stress accumulation. As the cycles progress, these effects may gradually saturate, resulting in a diminishing rate of increase in the maximum equivalent stress.

Due to the mismatch in the coefficients of thermal expansion between adjacent materials, alternating stresses may occur under temperature cycling loading, leading to fatigue failure. According to the simulation results of the sub-model, the maximum equivalent stress during thermal fatigue alternation occurs at the corners of the TSV and RDL contact interfaces. Therefore, this location is chosen as a critical point, where the maximum shear plastic strain amplitude is 1.45 × 10^−3^. The thermal fatigue life is calculated as $N_{f}=3.1708\times 10^{5}$ cycles, as detailed below:

(1) $$N_{f}=\frac{1}{2}{\left(\frac{\Delta \gamma_{p}}{2\varepsilon_{f}}\right)}^{1/c}=\frac{1}{2}\times {\left(\frac{0.00145}{0.6}\right)}^{-2.2173}\approx 3.1708\times 10^{5}\mathrm{cycles}$$

where $\gamma_{p}$ represents the fatigue toughness coefficient, and $\varepsilon_{f}$ represents the fatigue toughness exponent.

### 3.3. Stress Analysis of Copper Pillars Containing Defects in TSV

The copper pillars deposited within TSVs may develop defects, which can impact thermo-mechanical stress. This study simulated the electroplating process of copper pillars inside TSVs, aiming to replicate various types and shapes of defects occurring during copper filling. Subsequently, the obtained defects were introduced into the copper pillar structure, and a comparative analysis was conducted to assess the impact of electroplating defects on internal stress under temperature cycling loads.

**Figure 17:**
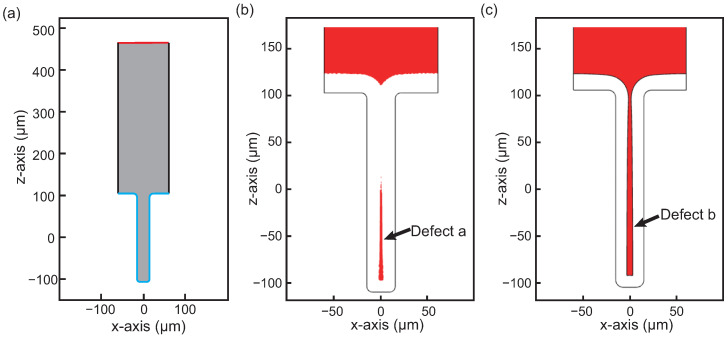
Simulation of defects in TSV: (**a**) geometry and boundary conditions, (**b**) defect a, and (**c**) defect b.

The model depicted in Figure 17a represents a Through Silicon Via with a filling diameter of 30 μm and an aspect ratio of 7:1. Within the model, the gray region symbolizes the copper sulfate plating solution, while the blue and red boundaries indicate the plating cathode and anode, respectively. The black boundaries delineate the insulation. A plating current is applied through the surfaces of the two electrodes.

Due to the edge effect of the electric field and electrolyte resistance in the via, the reaction potential at the top is larger than that at the via bottom, leading to pinch-off or void filling. Consequently, during the electroplating process of TSV filling, cavities are often generated. Two typical types of defects are identified, as demonstrated by the simulation outcomes in Figure 17b,c. Defect “a” exhibits a conical shape with a base diameter of 5.3 μm and a height of 105 μm, whereas defect “b” takes the form of a truncated cone with a top diameter of 6.3 μm, a bottom diameter of 3 μm, and a height of 181 μm. If the reaction potential at the top is much greater than that at the bottom via, sealing at the top occurs faster, leading to a tendency to produce defect “a”. Conversely, it is easier to generate defect “b”. These defect shapes closely resemble the typical void shapes observed during plating processes in TSVs, enhancing the model’s relevance and applicability [24,25,26,27,28,29].

**Figure 18:**
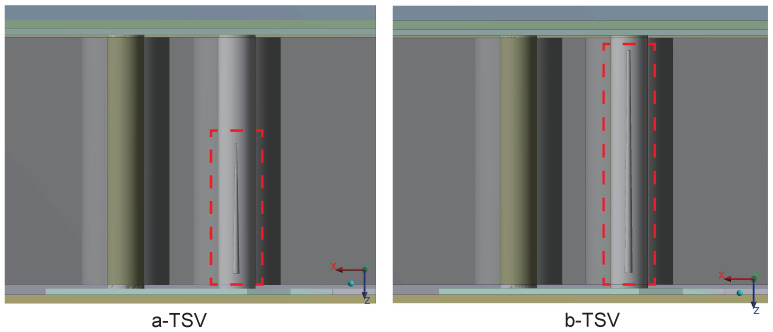
Model construction including electroplating defects, named a-TSV and b-TSV. The defects within the red dashed boxes.

Modifications were made to the copper pillar structure in the sub-model by introducing two types of plating defects internally. The model containing defect type a was labeled as a-TSV, while the model containing defect type b was labeled as b-TSV, as shown in Figure 18.

**Figure 19:**
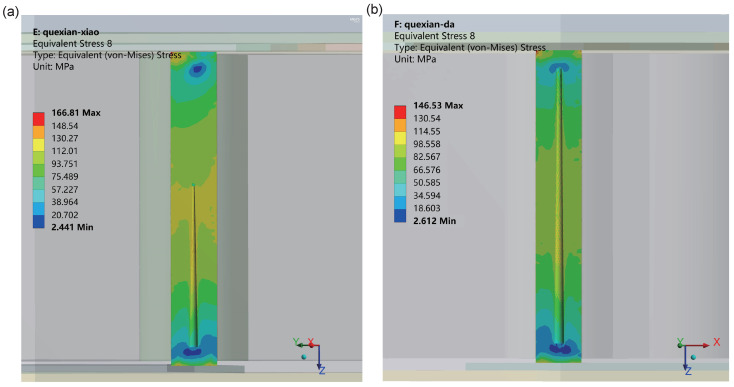
The stress distribution of copper with defects during the low-temperature stage in the last cycle: (**a**) defect a and (**b**) defect b.

In the low-temperature phase of the last temperature cycle, the distribution of equivalent stress in the copper pillars with defects inside the TSV is illustrated in Figure 19, where Figure (a) represents the presence of defect type a and Figure (b) represents the presence of defect type b. Upon comparing these two types of defects, it is observed that the maximum equivalent stress occurs at the corner of the TSV-RDL interface in both cases. The maximum equivalent stress on a-TSV is 166.81 MPa, whereas on b-TSV it is 146.53 MPa.

**Figure 20:**
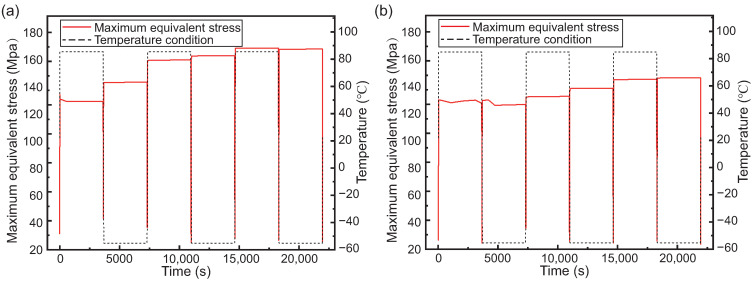
The maximum equivalent stress curve of copper with defects with temperature cycle: (**a**) defect a and (**b**) defect b.

Additionally, from Figure 20, it can be noted that for all of the temperature cycling process, the maximum equivalent stress on a-TSV consistently exceeds that on b-TSV. This discrepancy may be attributed to the shapes and sizes of the defects. In the case of defect type a, the region of stress concentration may be more pronounced, leading to a more significant stress concentration effect. In contrast, defect type b resembles a truncated cone, which may result in a more uniform stress distribution, thereby reducing the stress concentration effect. Furthermore, the larger size of defect type b allows for the release of stress in a greater number of regions.

## 4. Conclusions

This study conducts a thermal–structural analysis of a silicon interposer with complex structures used for 2.5D packaging, based on finite element simulation, including an investigation into the effects of electroplating defects. This study reveals stress and deformation asymmetry in TSVs due to supplementary layers. It also finds that stress stabilizes after multiple cycles, enabling accurate-reliability lifespan estimation. These findings provide theoretical guidance for reliability analysis under long-term complex temperature conditions and offer insights into potential void defects’ impact on reliability during manufacturing. The main findings are as follows: (1) During both high-temperature and low-temperature stages, the maximum equivalent stress of the copper pillars is located at the center of the overall model, indicating the most critical region. (2) Sub-model simulations reveal that under temperature loading, the deformation exhibits cyclic behavior, with the equivalent stress gradually accumulating. The cumulative effect of stress weakens with continuous loading, resulting in a thermal fatigue life of 3.1708 × 10^5^ cycles. (3) During copper electroplating, type b defects are found to reduce the resulting equivalent stress compared to type a defects. This indicates that sharp defects can cause stress concentrations, and that larger plating defects can reduce the equivalent stress. This study provides theoretical guidance and direction for subsequent process optimization. For example, a strain relief groove can be added in the center to improve the overall life. Moreover, adding a cylindrical cavity to the manufacture of copper pillars can be adopted to reduce stress, similar to the scheme of opening stress grooves on silicon substrates.

## Figures and Tables

**Table 1:** Internal structural dimensions of the interposer plate.

Structure	Layer’s Number	Layer’s Name	Material	Thickness
Top	First	Top silicon dioxide	SiO_2_	2 μm
	Second	Top RDL^1^	Cu	5 μm
	Third	Top PI^1^	PI	7 μm
	Forth	Top RDL^2^	Cu	5 μm
	Fifth	Top PI^1^	PI	7 μm
	Sixth	Top UBM	Cu	Thickness: 5 μm Diameter: 90 μm
Middle	—	Base layer	Si	200 μm
Bottom	First	Bottom silicon dioxide	SiO_2_	0.5 μm
	Second	Bottom PI^1^	PI	3 μm
	Third	Bottom RDL	Cu	5 μm
	Forth	Bottom PI^2^	PI	7 μm
	Fifth	Bottom UBM	Cu	Thickness: 5 μm Diameter: 240 μm

**Table 2:** Material parameters for simulation.

Material	ρ (g/cm^3^)	*E* (Mpa)	ν	α (1 × 10^−6^)	κ (W × m^−1^ × K^−1^)	*C* (J × kg^−1^ × K^−1^)
Cu	8.9	128	0.34	16.5	380	385
SiO_2_	2.648	75	0.17	0.5	1.3	1000
Si	2.329	169	0.26	2.49	156	713
PI	1.1	2.2	0.3	20	0.19	1100
Sn_63_Pb_37_	8.4	30.0	0.36	24.7	50	183
Substrate	3.9	380	0.27	5.8	27	900

**Table 3:** Multilinear isotropic hardening parameters.

Stress (MPa)	121	186	217	234	248
**strain**	0.001 ε	0.004 ε	0.01 ε	0.02 ε	0.04 ε

## Data Availability

Data are contained within the article.
